# A synthetic energy dataset for non-intrusive load monitoring in households

**DOI:** 10.1038/s41597-020-0434-6

**Published:** 2020-04-02

**Authors:** Christoph Klemenjak, Christoph Kovatsch, Manuel Herold, Wilfried Elmenreich

**Affiliations:** Institute of Networked and Embedded Systems, University of Klagenfurt, 9020 Klagenfurt, Austria

**Keywords:** Electrical and electronic engineering, Energy infrastructure

## Abstract

Research on smart grid technologies is expected to result in effective climate change mitigation. Non-Intrusive Load Monitoring (NILM) is seen as a key technique for enabling innovative smart-grid services. By breaking down the energy consumption of households and industrial facilities into its components, NILM techniques provide information on present appliances and can be applied to perform diagnostics. As with related Machine Learning problems, research and development requires a sufficient amount of data to train and validate new approaches. As a viable alternative to collecting datasets in buildings during expensive and time-consuming measurement campaigns, the idea of generating synthetic datasets for NILM gain momentum recently. With SynD, we present a synthetic energy dataset with focus on residential buildings. We release 180 days of synthetic power data on aggregate level (i.e. mains) and individual appliances. SynD is the result of a custom simulation process that relies on power traces of real household appliances. In addition, we present several case studies that demonstrate similarity of our dataset and four real-world energy datasets.

## Abstract (summary)

**Table:** 

Measurement(s)	energy
Technology Type(s)	computational modeling technique
Factor Type(s)	appliance •operational state
Sample Characteristic - Environment	residential building

Machine-accessible metadata file describing the reported data: 10.6084/m9.figshare.11940324

## Background & Summary

Load monitoring is vital for effective and accurate energy monitoring in buildings. Detailed insights can empower further research, help streamlining processes, and improve a building’s energy efficiency^1^. Introduced in^2^, Non-Intrusive Load Monitoring (NILM) techniques serve to break down a building’s aggregate energy consumption to identify active appliances and also to provide diagnostic information. Extensive reviews can be obtained from^3^ and^4^. NILM can be considered as Machine Learning problem. As such, it requires datasets to train models, to conduct performance evaluation, to evaluate the benefit in real scenarios, and also to perform benchmarking on a common basis. In case of NILM, ground-truth data on aggregate and appliance-level energy consumption are crucial^4^.

Traditionally, NILM scholarship relies on energy consumption datasets. Such datasets usually contain information on energy consumption on aggregate level (monitored at the mains) and individual loads, which is provided by plug-level meters. Energy consumption datasets are the outcome of measurement campaigns in buildings or industrial facilities, which require expensive measurement equipment, bring bureaucratic burdens, and are time-consuming activities^5^. As a viable alternative, the idea of generating synthetic data gain momentum recently. The main motivation behind generating synthetic datasets is to reduce costs for measurement campaigns and save valuable work hours. Instead, custom simulators provide energy consumption datasets on-demand and in contrast to real datasets, without limitations on measurement periods. Furthermore, real datasets suffer from missing readings (gaps), misaligned timestamps, and corrupted data as a result of sensor miscalculation or malfunction^6,7^. Synthetic data does not show such issues.

With SynD, we present a synthetic energy consumption dataset for Non-Intrusive Load Monitoring (NILM) with focus on the residential sector. SynD provides 180 days of a simulated household with 21 household appliances. We derive custom appliance models from the outcome of our measurement campaign in two Austrian households and by applying a modelling approach similar to^8^ and^9^. As it is shown in the evaluation, the household simulated in SynD can be associated with a relaxed lifestyle of a single person or a young couple. We implemented a dataset generator that utilises our custom appliance models to simulate one household for given input parameters such as sampling rate and duration. As traditional energy consumption datasets, SynD provides aggregate power readings as well as power readings of individual household appliances. Furthermore, our dataset complies with the majority of suggestions for energy datasets, which were presented in^10^. For instance, we release SynD in two different versions: Besides the widely-used CSV format, we also provide a HDF5 version of SynD that is fully compatible with NILMTK^11^, a toolkit for reproducible NILM experiments with state-of-the-art algorithms^12^.

To the best of our knowledge, there exist three major contributions on synthetic dataset generation with regard to NILM: AMBAL^8^, SmartSim^13^, and SHED^14^.

The Automated Model Builder for Appliance Loads (AMBAL), presented in^8^, extracts appliance models from real datasets. These models consist of sequences of parametrised signatures and are used by a trace generator to simulate a real household. Since the creators of AMBAL haven’t released a dataset, we report insights provided by^8^. Besides a statistical analysis of commercial and residential energy datasets, the authors of^14^ released SHED (https://nilm.telecom-paristech.fr/shed/), a synthetic dataset with focus on commercial buildings. SHED is generated by a custom algorithm that simulates current and power readings for several buildings. We draw comparisons on the basis of provided power consumption data in SHED. SmartSim is a device-accurate smart home energy trace generator. This simulation framework utilises device energy models and device usage models to simulate a household. SmartSim leverages its modelling methodology from empirical characterisation studies presented in^9^. Device models in SmartSim build on energy data from Smart*, a real-world energy dataset^15^. To compare SynD and SmartSim, we consider the latest version on Github (https://github.com/klemenjak/smartsim/tree/master/house_1).

We summarise key differences between related work and our contribution in Table 1. SynD provides 180 days of a simulated household that consists of 21 appliances. We provide aggregate and submeter readings at a rate of 5 Hz, which is suspected to be suitable for low-frequency NILM investigations, as a recent study on data requirements for NILM claims^16^. Besides energy data, we provide an extensive amount of metadata in the NILM metadata format^17^.

**Table 1:** Comparison of existing synthetic energy datasets.

	AMBAL	SmartSim	SHED	SynD
Appliances	14	25	66	21
Duration	n/a	7 days	14 days	180 days
NILMTK format	n/a	No	No	Yes
Released data	No	Yes	Yes	Yes
Sampling Rate	1 Hz	1 Hz	0.033 Hz	5 Hz
Scope	residential	residential	commercial	residential

## Methods

In this section, we depict the methods applied to create the synthetic energy consumption dataset (SynD). First, we report on a measurement campaign that was conducted in real households in Carinthia, a province of Austria. Second, we explain how our approach categorises household appliances to group them according to their energy consumption behaviour. Finally, we describe in detail our dataset generation approach.

### Measurement campaign & appliance categories

**Fig. 1:**
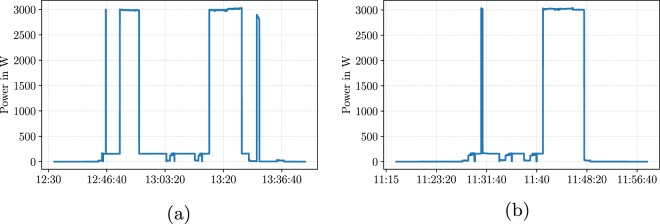
Power consumption patterns of the dishwasher in SynD: (**a**) pattern of programme A (**b**) pattern of programme B.

During a measurement campaign in two Austrian households, one in Klagenfurt and one in Villach, we monitored 21 electrical household appliances. The main goal of the measurement campaign was to record representative power consumption patterns of those 21 appliances, where a power consumption pattern is represented by the shape of the power consumption over time for a single operation^18^. Table 2 summarises monitored appliances, their manufacturer, and the number of recorded patterns during the campaign. For household appliances with a wide variety of operational programmes or adjustable settings such as temperature or intensity, we recorded power consumption patterns of the most-frequently-used options. Figure 1 shows recorded power consumption patterns for two programmes of a dishwasher. Although both power consumption patterns refer to the same device, we can observe a clear difference in terms of shape, length, and energy consumption between the two patterns.

**Table 2:** Household appliances in SynD.

ID	Household appliance	Manufacturer	Patterns	Category
2	Fridge	Bomann	1	Periodical
3	Dishwasher	Bosch	3	Multi
4	Electric heater	Ningbo Elect.	2	Multi
5	Washing machine	Miele	2	Multi
6	Toaster	Philips	3	Multi
7	Fan	CasaFan	2	Multi
8	Microwave	Siemens	3	Multi
9	Iron	Moulinex	2	Multi
10	Hot air gun	Thermo Elect.	2	Multi
11	Router	Linksys	1	Constantly-On
12	Coffee machine	DeLonghi	3	Multi
13	TV	Panasonic	2	Multi
14	Printer	HP	2	Multi
15	Laptop computer	Lenovo	2	Multi
16	Lamp	TaoTronics	1	Single
17	Gaming PC	Acer	2	Multi
18	Pocket Radio	Schneider	1	Single
19	Monitor	DELL	1	Single
20	Electric oven	Severin	1	Single
21	Hair dryer	Philips	1	Single
22	Water kettle	CLA Tronic	1	Single

**Fig. 2:**
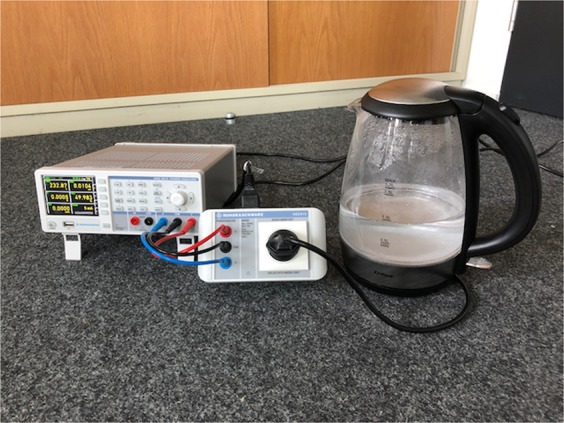
Reenactment of our measurement setup.

As data logger, we used a Rohde & Schwarz HMC8015 power analyzer, which provides compliance with IEC 62301, EN 50564, and EN 61000-3-2. Table 3 summarises the main specifications of this device. With a measurement accuracy of 0.05% of reading and a temporal resolution of 100 ms, the measurement device meets the instrumentation requirements for energy datasets suggested in^10^. In conjunction with a socket adapter, the HZC815-EU EU connector, we attached the measurement device to one electrical appliance a time. Figure 2 depicts how measurements were conducted. We gathered the outcome of our measurement campaign in form of CSV files, which contain active-power readings with a sampling interval of 100 ms.

**Table 3:** Specifications of the HMC8015 power analyzer.

Specification	Description
A/D converter resolution	16 bit
Measurement accuracy	0.05 % of reading
Power range	50 *μ*W to 12 kW
Physical quantity	active power in W
Resolution of output data	100 ms
Sampling frequency (waveform)	500 Hz

One way of categorising appliances is through the number of operational states^3^. In our considerations, we focus on specific time windows of power consumption rather than on single operational states. Inspired by the empirical characterisation in^9^, the automated model builder for appliance loads^8^, and the concept of predictability of power consumption patterns in^18^, we defined four appliance categories: constantly-on, periodical, single pattern, and multi pattern.

- *Constantly-On*: Appliances of this group consume energy without any downtime. In our dataset, an example of such an appliance is the WiFi router, which operates continuously.
- *Periodical*: We refer to appliances, which run autonomously and have a recurring consumption pattern, as periodical appliances. A common example for periodical appliances are fridges. Fridges operate autonomously and have predictable duty cycles.
- *Single pattern*: The vast majority of household appliances do not operate autonomously i.e. they require a user either to operate or to start a specific programme. From that follows that such appliances are activated by a user, perform a specific task, and turn off or are turned off after completion of that task. The group of single-pattern appliances considers appliances with a single power consumption pattern. For instance, we can observe a similar power consumption pattern during every operation for water kettles. External factors such as the filling level of the kettle influence the length of the pattern to a certain degree but the main characteristics of the pattern, such as peak consumption and shape, can be predicted fairly well.
- *Multi pattern*: Appliances of the multi-pattern category offer several modes of operation with distinct power consumption patterns. Examples for multi-pattern appliances are dishwashers, washing machines, and electric heaters. Patterns of such appliances not only differ in length but also show distinct process steps. From that follows, that appliances perform different tasks during these programmes that can lead to completely different power consumption patterns. Figure 1 shows power consumption patterns of two different programmes of the dishwasher in SynD. We observe clear differences between the two patterns. Therefore, we want to emphasise the importance of considering multiple consumption patterns to better model such appliances.

An overview of household appliances and associated categories in our dataset is provided in Table 2. The difficulty of categorising household appliances lies in the extraction of consumption patterns and the predictability of appliance usage as well as duration of appliance usage^18^. While some appliances such as dishwashers are designed to have clear programmes of operation with a predictable end time, it is challenging to identify the most appropriate power consumption pattern for user-controlled appliances such as hair dryers and microwave ovens. We addressed this issue by incorporating expert knowledge into our measurement campaign, which is a result of studies related to a personalised feedback system for energy management in households^19^ and conclusions drawn from the outcome of a measurement campaign in Austrian households^20^. Based on this knowledge, we adapted the residents behaviour during measurements, i.e. appliance usage, in a way to produce as representative consumption patterns as possible.

### Dataset generation

SynD is the result of a simulation process that relies on power consumption patterns of existing household appliances in two Austrian households. We provide detailed insights on the simulation process following a top-down approach. We begin with the big picture of our implementation and conclude with details on dynamic placing and interpolation of consumption patterns.

**Fig. 3:**
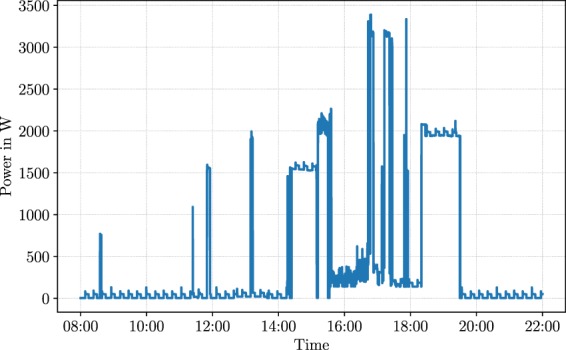
One day in the life of SynD.

In principle, the simulation follows a straightforward procedure, as Box 1 outlines. Parametrised by a set of input parameters, we simulate the power consumption of one imaginary household day by day. In our simulation approach, days are defined to be independent observations i.e. the energy consumption of one day does not influence the energy consumption of the next day. While a real household might show some correlations of appliance usage between subsequent days or week days we decided for a simple model assuming independent days, since this effect is hard to characterise based on existing data and is not very relevant for current load disaggregation algorithms. For every day in our simulation, we obtain the power consumption of selected household appliances individually. As per default, we consider all 21 appliances. In addition to individual power readings of appliances, we also obtain the aggregate power consumption of the household by accumulating the individual power readings of appliances. Figure 3 shows the aggregate power signal for one day. Aggregate power signals are particularly interesting for applications such as Non-Intrusive Load Monitoring (i.e. load disaggregation) and energy forecasting.

As soon as the simulation process finishes, the obtained dataset is either saved to a HDF5 file following the NILMTK data format^11^ or compressed to a ZIP archive. In case of the ZIP archive, this archive contains metadata as well as 22 CSV files (one file per appliance plus one file for the aggregated power).

Our simulation approach assumes that household appliances don’t alter their behaviour due to operation of other present appliances i.e. appliances operate independently. We simulate the power consumption behaviour of appliances individually and neglect any correlations between them, which was identified as a necessary step to simplify the modelling problem. Appliance simulations in SynD share a set of input parameters: sampling interval, duration and power type. As per default, the simulator generates a dataset with a duration of 180 days and a sampling interval of 0.2 s. The vast majority of low-rate NILM datasets provide either active power readings or apparent power readings^5^. Also, active power readings are used for billing in real energy grids. For this reason, the emphasis of our simulation process is on active power. Our approach simulates the power consumption of appliances day by day. To generate data for a new day, the simulation process follows three steps, which we will discuss in detail:

1. Selection of a power consumption pattern from templates
2. Interpolation or resampling of the selected pattern
3. Identification of the time of usage and insertion of the selected pattern

The first step of simulating the power consumption of an appliance in SynD is to select a power consumption pattern for the current day of simulation. As already pointed out, we defined four different appliance categories: constantly-on, periodical, single-pattern and multi-pattern. The category of an appliance decides on how a power consumption pattern is selected during the simulation:

- For appliances of the *constantly-on* category, such as the router, the simulator loads the power consumption pattern recorded during the measurement campaign and successively inserts this pattern until data for one day is generated.
- Appliances such as fridges show a *periodical* power consumption behaviour. For such appliances, we recorded multiple operational cycles during the measurement campaign. To mimic real periodical appliances, the simulation loads the recorded data and inserts this sequence of power consumption patterns until data for one day is generated.
- For appliances of the *single-pattern* category, the simulator selects the one power consumption pattern recorded during the measurement campaign.
- We incorporate several *multi-pattern* appliances in SynD, as Table 2 shows. For appliances of this category, the simulator randomly selects one of the recorded patterns, where all patterns are equally likely to be selected.

For appliances of the categories constantly-on and periodical, the simulation is completed after the first step. This is because our approach mimics the real-world behaviour of constantly-on and periodical appliances by repeatedly inserting data that was recorded during the measurement campaign and therefore, no further processing is required. For example, we expect fridges to show a strong periodical behaviour without any noticeable deviations from it (unless the fridge is open for a considerable duration or warm food has been put in). In contrast to that, simulation of single and multi-pattern appliances require more wide-ranging processing strategies in order to better mimic their true behaviour. For instance, after selecting a power consumption pattern for single and multi-pattern appliances, we introduce a random variable with a uniform probability distribution, which decides whether or not to ignore the selected pattern. In this way, we randomly ignore the outcome of the pattern selection since in real households, residents rarely use all of their appliances on a daily basis. Instead of the selected power consumption pattern, we insert a Null vector for that day in case the random variable prompts the simulation to ignore the pattern. For every single and multi-pattern appliance, we defined a unique probability distribution. The probability distributions have been obtained from the appliance utilisation in GREEND^20^, an energy consumption dataset that is the outcome of measurement campaigns in several Austrian and Italian households.

In principle, appliances can be divided into two main groups. The first group defines clear programmes, which result in predictable power consumption patterns. Wide-spread examples for this group are dishwashers and washing machines. Such appliances offer a set of different washing programmes, which result in more or less the same power consumption pattern. For this group of appliances, our simulator does not perform any manipulation to the selected power consumption pattern. In contrast to the first group, there exists a big variety of electrical appliances without unique or pre-defined programmes. For instance, hairdryers, vacuum cleaners, microwave ovens, water kettles and electric heaters belong to this group^18^. These appliances are either actively controlled by residents or strongly depend on individual user settings. Furthermore, such appliances show considerable variations in terms of daily energy usage. To mimic this behaviour, we implemented a special interpolation policy for this second group of appliances: First, the simulator checks if interpolation is required for the selected appliance i.e. to what category an appliance belongs. If there is a need for interpolation, then the simulator draws a random number from a uniform distribution. The parameters of the uniform distribution depend on the appliance and are listed in Table 4. We derived those parameters by analysing existing datasets and estimating common lower and upper duration of usage per appliance. The obtained sample defines the length of the power consumption pattern after interpolation i.e. the duration. Finally, the simulator applies interpolation to alter that specific power consumption pattern. In this way, we add new samples to the pattern or remove samples from the pattern, depending on the targeted length of the pattern.

**Table 4:** Pre-defined parameters for dynamic placing and interpolation: range of the mean for start time, standard deviation of the start time, variation of usage duration.

Appliance	Range of mean *μ* [time of day]	Std. deviation *σ* [min]	Interpolation [min]
Toaster	08:00–09:30	15	—
Washing machine	14:00–16:45	60	—
Dishwasher	12:30–16:40	90	—
Fan	12:30–16:40	145	17–84
Heater	18:00–19:00	30	50–167
Hot air gun	11:00–12:30	30	3–7
Iron	13:30–15:15	30	40–100
Microwave	16:30–17:45	15	2–5
Radio	08:30–09:30	30	15–35
Water kettle	11:30–17:00	30	3–7
Hairdryer	07:45–16:45	30	4–8
Electric oven	08:00–17:15	60	5–15
Monitor	14:00–16:45	1	20–100
TV	15:15–19:00	1	35–250
Printer	09:45–19:30	1	1–15
Coffee machine	08:20–15:15	1	—
Laptop	11:00–19:30	1	15–85
Lamp	16:40–21:00	1	15–50
Gaming PC	14:00–19:30	1	80–167

**Fig. 4:**
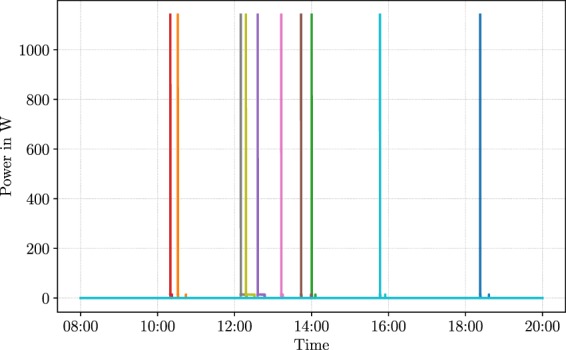
Variation of the power-on time for the printer for ten different days.

Residents distinguish themselves by special habits and individual daily routines. On a household-wide level, this may lead to certain time windows with higher energy consumption i.e. residential rush hours. However, assuming that appliances always operate at the exact same time of the day represents a misleading modelling assumption. For this reason, a reasonable level of timing variation has to be introduced to appliance simulations i.e. appliance usage has to be shifted within reasonable time windows. We approach this issue by spreading out the use of household appliances during the day. We implemented a random placing mechanism that randomly selects power-on times of appliances from pre-defined time windows. Those time windows were defined for single and multi-pattern appliances and are summarised in Table 4. We derived those time windows from studies related to a personalised feedback system^19^ and a measurement campaign in Austrian households^20^. We define one uniform distribution per appliance based on those time windows. During the simulation of an appliance, we draw a sample from its associated uniform distribution, e.g. we obtain a sample between 11:30 and 17:00 for the water kettle. In conjunction with a pre-set value for the standard deviation *σ*, this sample serves as mean *μ* to parametrise a normal distribution. Next, we draw a sample from that normal distribution to obtain the power-on time of the appliance. Our simulator ensures that the power-on time of an appliance cannot lie on the following day. This way, we are confident that the power consumption of one day cannot influence the following day. For example: In case of the dishwasher, we draw a sample from its associated uniform distribution to obtain a time between 12:30 and 16:40. Let’s assume we obtain the time *13:45*. As next step, we convert this time to the number of minutes since midnight (825 min). This number serves as the mean of a normal distribution with a standard deviation of 90 min, as Table 4 reports. To obtain the power-on time of the dishwasher, we draw a sample from the normal distribution ${\mathcal{N}}(\mu =825\ {\rm{m}}{\rm{i}}{\rm{n}},\sigma =90\ {\rm{m}}{\rm{i}}{\rm{n}})$. The obtained sample defines the starting time of the dishwasher for the current day of the simulation. Figure 4 illustrates the result of our random placing strategy for another common appliance: a printer. The plot shows ten simulated days for the printer. We observe a clear spread of the patterns during the day with different distances between the inserted patterns. We perform this special placing method in order to increase the probability of obtaining different starting times for appliances even if we draw the same starting times for two appliances in the first step. In this special case, the normal distribution in the second step would still provide distinct starting times for those two appliances. Avoiding identical switching times of appliances is said to be an important detail in certain research problems. For instance, the Switch Continuity Principle represents an essential assumption in Non-Intrusive Load Monitoring (NILM)^21^ and must not be neglected. By deriving the power-on times in a nested manner and through utilisation of several probability distributions, we aim to achieve strong compliance with the Switch Continuity Principle (SCP) in our dataset.

Our implementation of SynD builds on random number generators provided by the Numpy package. Those generators support initial seeding to foster repeatability of simulations. As generator for discrete uniform distributions, we selected *randint*. This function draws integers from a half-open interval [a, b) following a probability density function (PDF):

1 $$f(x)=\left\{\begin{array}{cl}\frac{1}{b-a} & \,for\,x\in [a,b)\\ 0 & \,otherwise\,\end{array}\right.$$

For normally-distributed samples, we incorporate the generator *normal*. Samples provided by this generator follow the probability density function (PDF):

2 $$f(x)=\frac{1}{\sqrt{2\pi {\sigma }^{2}}}{e}^{-\frac{{(x-\mu )}^{2}}{2{\sigma }^{2}}}$$

The actual shape of the PDF is parametrised by the mean *μ* and the standard deviation *σ*.

Box 1 The simulation process in SynD.

## Data Records

With SynD, we release an energy consumption dataset that consists of synthetic data. For a duration of 180 days, we simulated a household in Austria, where the emphasis of simulations was on consumption of electrical energy. The utilised appliance models build on data that was recorded during a measurement campaign in real households. This data can be found in the archive *appliance_traces.zip*. Table 5 summarises key properties of SynD. The current version, published in a figshare data repository^22^, contains simulated active power readings of 21 appliances. More information on appliances embedded in SynD can be obtained from Table 2. This version of SynD comes with a sampling interval of 0.2 s. Beyond power readings, we provide detailed metadata on appliances and an HDF5 version of SynD, which is compatible with the Non-Intrusive Load Monitoring Toolkit (NILMTK)^11^.

**Table 5:** Basic information on SynD.

Specification	Description
AC power types	active power in W
Compatible to NILMTK	Yes
Duration	180 days
File format	CSV and HDF5
Number of appliances	21
Number of households	1
Origin of ground-truth	Austria
Sampling interval	0.2 s

The initial release of SynD comprises four files, as Table 6 lists. Inspired by suggestions made in a recent paper on energy datasets^10^, we release SynD in two different formats: CSV and HDF5. The CSV version of SynD can be obtained from *SynD_CSV.zip*. This archive consists of 22 CSV files, where one CSV file contains the power time series of one appliance a time. The filename indicates to what appliance the data is associated with. Box 2 shows the top of the file 1 .csv, which summarises the mains power consumption over time. Human-readable timestamps serve as index and tabulators as delimiters in all CSV files of our release.

**Table 6:** Files associated with SynD.

File Name	Description
appliance_labels.yml	Explains mapping of IDs and appliances.
appliance_traces.zip	The power traces used to create appliance models.
metadata.zip	Contains metadata of dataset, meters and appliances.
dataset_generator.zip	The generator used to create SynD.
SynD.h5	The NILMTK version of SynD.
SynD_CSV.zip	The CSV version of SynD.

The file *appliance_labels.yml* includes a Python dictionary that explains the mapping of CSV filenames and appliances in SynD. The HDF5 version of SynD can be obtained from *SynD.h5*. The zip file *metadata.zip* offers comprehensive metadata on the dataset, measurement devices (HMC8015), and all 21 household appliances. Across all metadata files, we apply the metadata schema presented in^17^. We selected this metadata schema (https://github.com/nilmtk/nilm_metadata) because of its great acceptance within the NILM community. In Box 3, we show metadata for the coffee machine as an example. To the best of our abilities, we collected information on the type, nominal power consumption and manufacturer for all appliances. The metadata files provided alongside the dataset are meant to serve as important resources for future investigators. We provide information on appliance-specific information, details on measurement devices, general remarks to our dataset, and references to further resources.

The methods section provides information on our dataset generation approach in form of clear step-by-step instructions on an abstracted level so that our approach can be understood without digging deep into source code. However, to give experts better insights into our simulation approach, we release the first public version of our dataset generator along with SynD. The archive *dataset_generator.zip* contains an executable version of our generator with pre-defined settings. We would like to emphasise that future versions of this toolkit will be published on our Github repository.

**Fig. 5:**
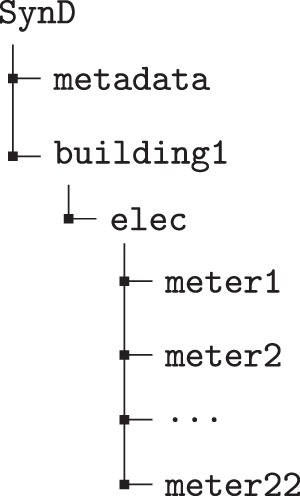
NILMTK-DF format hierarchy for SynD.

The Non-Intrusive Load Monitoring Toolkit, NILMTK, enjoys a high reputation in the NILM research community. Introduced in^11^, it provides functionalities to perform dataset analysis and aims to enable benchmarking of load disaggregation algorithms. Recent contributions, presented in^12^, extend the toolkit by introducing new APIs for disaggregation and experiments. To lower the entry barrier for NILMTK users, we provide a NILMTK-compatible version of our synthetic dataset. This version of SynD uses the NILMTK-DF file format^11^. This allows seamless integration into the toolkit and therefore, easy access to power readings. Figure 5 shows the hierarchical model of the SynD household. SynD contains one energy meter group (elec) that consists of 22 meters. Meter1 represents the mains, i.e., aggregate power consumption of the household. Meter2 to meter22 contain power readings of one appliance per meter. In this version of SynD, power readings are stored as Pandas DataFrames and indexed by human-readable timestamps. We demonstrate how to access and plot data from SynD using NILMTK in Box 4.

Box 2 The head of file 1 .csv. 1 2019-09-29 00:00:00.000 3.842

2 2019-09-29 00:00:00.200 3.842

3 2019-09-29 00:00:00.400 3.832

4 2019-09-29 00:00:00.600 3.840

5 ...

Box 3 Metadata of the coffee machine. 1 # coffee_machine.yaml

2 rooms:

3 - B10.2.014

4 meter_model: HMC 8015 Power Analyzer

5 appliance:

6   type: Coffee machine

7   components:

8     type: ESAM 04.120 Magnifica S

9 nominal_consumption:

10  bias: 240

11  current: 10

12  frequency: 60

13  power: 1450

14 manufacturer: DeLonghi

15 year_of_manufacture: 2011

Box 4 Sample code for NILMTK. 1  from nilmtk import DataSet

2  import matplotlib.pyplot as plt

3  SynD = DataSet (’SynD.h5 ’)

4  elec = SynD.buildings [1].elec

5  print (elec)

6  plt.plot (elec.mains ().power_series_all_data ())

7  plt.ylabel (’Power in W’)

8  plt.xlabel (’Time’)

9  plt.grid (color=’0.75’, linestyle =’-.’, linewidth=0.5)

10 plt.title (’One day in the life of SynD’)

## Technical Validation

Real-world energy datasets are the outcome of measurement campaigns in households and/or industrial facilities with special attention to not disrupt daily routines within the monitored household so that the recorded data resembles reality as best as possible^5^. In this section, we present analyses and case studies that signal strong similarity between our synthetic energy dataset SynD and real-world energy consumption datasets. In our studies, we use data from multiple households embedded in four common energy consumption datasets: DRED^23^, ECO^24^, REFIT^25^ and UK-DALE^26^. We paid attention to select households that are commonly used in related work. An extensive list of available energy datasets can be obtained from^5^. By assessing the similarity between real and synthetic data, we demonstrate that SynD represents a valid energy dataset. Our validation studies focus on two aspects of energy consumption datasets:

1. *Aggregate consumption*: We study differences in the energy consumption of households on an aggregate level (i.e. smart meter data)
2. *Power consumption of single appliances*: We study appliance usage in households and analyse similarities between power readings from real households and SynD

### A comparison of households

A household’s aggregate power signal, obtained from a smart meter, can provide deep insights into daily routines of residents, individual habits, and present appliances such as heat pumps^27^. Smart meter data can also be used to predict energy consumption of households^28^. The authors of^29^ provide a comprehensive review of smart meter data analytics. With regard to a synthetic energy dataset, the question arises how well such a simulated time series resembles aggregate power series of real households. For this reason, we present a study that compares aggregate power readings of SynD and readings obtained from real households. For the duration of forty days, we extracted the aggregate power signal from house 1 in DRED, house 1 and 2 in ECO, house 1 and 2 from REFIT, and house 1, 2 and 5 from UK-DALE.

**Fig. 6:**
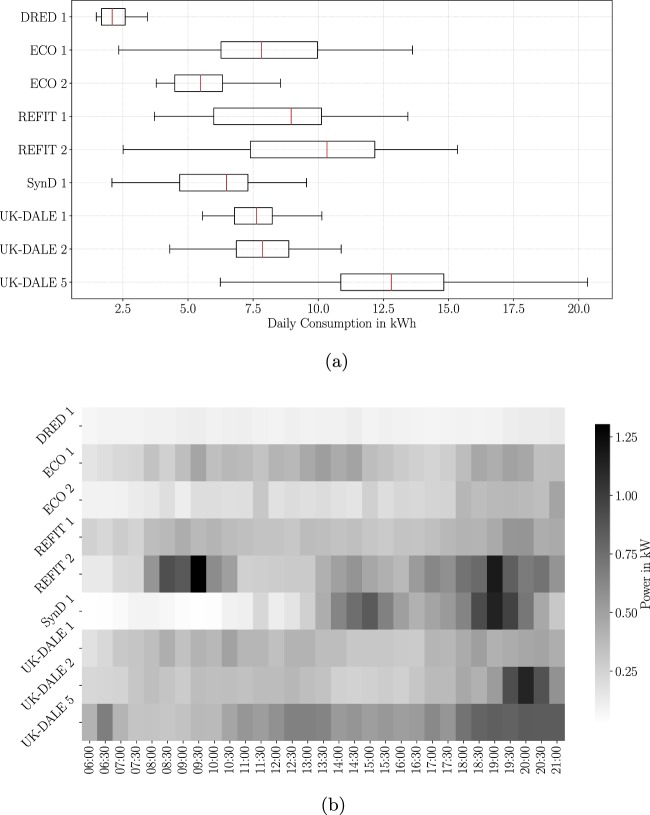
A comparison of aggregate power data: (**a**) variation of daily energy consumption for forty days (**b**) heatmap for average load profiles of forty days.

As a first step, we computed the daily energy consumption of those households for forty consecutive days. The boxplot in Fig. 6a gives insights on how much the daily energy consumption varies across the observed households. With 2.09 kWh, we observe the lowest median energy consumption in house 1 of DRED, whereas house 5 of UK-DALE shows the highest median energy consumption with 12.80 kWh. The household composed of synthetic data, SynD, ranks in the middle of observed households with 6.47 kWh. Nearest neighbours of SynD are house 1 of UK-DALE 7.62 kWh and house 2 in ECO 5.47 kWh. Furthermore, the box associated with SynD shows an intermediate box size, which indicates that the average deviation from the median lies in a realistic range compared to a narrow box for DRED and a rather wide box for house 2 of REFIT. To summarise the findings presented by Fig. 6a: The results of our first study indicate that based on the average daily energy consumption, the synthetic household in SynD appears to be very similar to a real household. Neither does the daily energy consumption of SynD focuses on a narrow interval nor we observe outliers during our observation period of forty days.

With regard to the energy consumption of single days, it is important to examine at what time of the day households consume the largest amount of energy. For a synthetic dataset, it is important to demonstrate that appliance usage is assigned to realistic time windows. For instance: The average person would not classify dishwasher usage in the middle of the night as a common event, though rare exceptions may apply. In our second validation study, we derived the average load profile of nine households for a duration of forty consecutive days, eight of them being real households and the remaining one the household embedded in SynD. We illustrate those average load profiles with the help of a heatmap. The heatmap in Fig. 6b divides the load profiles into time slots with a duration of 30 min. For every time slot, we plot the average power consumption during that time window. For many households, we observe strong similarities between ECO 1, REFIT 1, and UK-DALE 1. The households ECO 2 and DRED 1 show considerable lower levels of power consumption for most times of the day compared to other households in this study. Particularly noticeable are apparent special characteristics of some households: In REFIT 2, we identify a considerable high level of power consumption in the morning, which has power consumption levels similar to UK-DALE 2 and SynD 1 during late evenings. During the evening as well as late evening, we identify strong similarities between the real households UK-DALE 2, REFIT 2 and the simulated household SynD 1. In general, SynD closely resembles real households during the second half of the day. However, we identify considerably lower levels of power consumption in SynD during the morning, which rather resemble levels observed in the households ECO 2 and DRED 1.

We suspect two independent causes to account for these differences: First, our dataset does not contain any white goods with substantial power consumption such as common electric stoves. As a consequence, activities during breakfast time such as preparing ham and eggs is not reflected in the energy consumption during the morning. Also, our dataset does not include electric water heaters, which would operate in the morning. Second, load profiles are strongly influenced by the lifestyle and daily routines of residents. Families, senior citizens, adults, and young adults all distinguish themselves in their wake-up time as well as the duration they stay inside their homes in the morning. Simulations related to SynD were implemented by young adults and students for the large part. While most tasks like measuring a device are straightforward and have been meticulously performed, we have to assume that some design decisions for example on selecting a given device or on looking up reasonable schedules in other datasets might have been by the students’ own interpretation of a normal lifestyle. However, evaluation of the dataset quality in terms of comparison to other real datasets has been done independently to avoid students’ designing simulations that look realistic to them. As we can obtain from the heatmap, there is little power consumption during the morning, medium consumption during the afternoon and rather large consumption during the late evening. To summarise, the household simulated in SynD can be associated with a rather laid-back lifestyle of a single person or a young couple having little energy consumption before noon and use their appliances during the afternoon and night time. Furthermore, it should be pointed out that Fig. 6b shows that there is not a single time window for SynD with quixotic levels of power consumption i.e. all time windows of our synthetic dataset show reasonable power consumption levels.

### Similarity of household appliances

An integral part of our implementation of a synthetic energy consumption dataset is the simulation of individual load signals i.e. simulation of single appliances. Our simulation approach builds on power consumption patterns that were recorded during a measurement campaign in real households. During the simulation of SynD, we manipulate, resample, and interpolate those patterns according to our random placing policy. As a result of this complex simulation, the question arises how similar those simulated appliances are to real appliances. We demonstrate the validity of our approach by means of two studies: In the first study, we compare the energy consumption of simulated appliances to appliances monitored in real-world energy datasets for a time window of forty days. In the second study, we apply statistical measures to examine similarities of SynD and other datasets.

For a duration of forty consecutive days, we computed the energy consumption of dishwashers, fridges, washing machines and water kettles for multiple households of DRED, ECO, REFIT, SynD, and UK-DALE. Where possible, we selected data from the same season to achieve a fair comparison. It should be noted that we apply the same time window as in the previous study i.e. forty days per household. Table 7 lists the energy consumption per appliance. We mark those households that don’t contain a respective appliance type as not available (n/a). We notice that the energy consumption of appliances differs significantly between the observed households. For example, the dishwasher in REFIT 1 consumed 7.75 kWh over a period of forty days, whereas the dishwasher in REFIT 2 devoured 43.19 kWh. Similarly, we observe an energy consumption of 32.27 kWh for the washing machine in UK-DALE 5, whereas in house 2 of the same dataset, we identify merely 2.28 kWh. These differences in energy consumption can have various causes. For instance, the energy consumption of appliances strongly depends on the number of residents, their habits, family situation, etc. As a result, common household appliances such as dishwashers and washing machines may operate more frequently in households with larger families. Second, appliances of the same kind but different device model may differ substantially in terms of energy consumption. As a consequence, two different appliances that are built to serve the same physical task may require different levels of energy consumption to complete that specific task. Whatever the origin of different levels of energy consumption may be, we observe similarities between certain groups of dishwashers, washing machines, and water kettles. Interestingly, water kettles in British datasets (REFIT & UK-DALE) seem to consume considerably more energy over those forty days than their Continental-European counterparts (DRED & ECO) in this study. More studies on electric kettles can be found in related work, where researchers present studies on usage patterns and discuss potentials for energy savings^30^. We observe that the water kettle in SynD shows a similar energy consumption level as the kettles in DRED and ECO. As concerns the simulated household appliances of SynD, we observe that their energy consumption ranks either in the upper third or in the middle of energy consumption. As a consequence of this ranking, we speculate that our simulation process generates a sufficient amount of patterns.

**Table 7:** Energy consumption of selected household appliances for forty days.

Dataset	House	Energy Consumption in kWh			
		Dishwasher	Fridge	Washing machine	Water Kettle
DRED	1	n/a	28.45	4.60	2.45
ECO	1	n/a	16.19	22.04	4.27
ECO	2	15.94	19.70	n/a	2.39
REFIT	1	7.75	15.25	10.12	n/a
REFIT	2	43.19	28.19	14.15	23.06
REFIT	8	n/a	7.80	20.91	16.17
*SynD*	*1*	*26.52*	*16.98*	*24.92*	*4.48*
UK-DALE	1	12.57	30.71	21.04	11.71
UK-DALE	2	7.69	5.49	2.28	34.46
UK-DALE	5	13.09	30.85	32.27	0.00

#### Statistical similarity of appliances

**Fig. 7:**
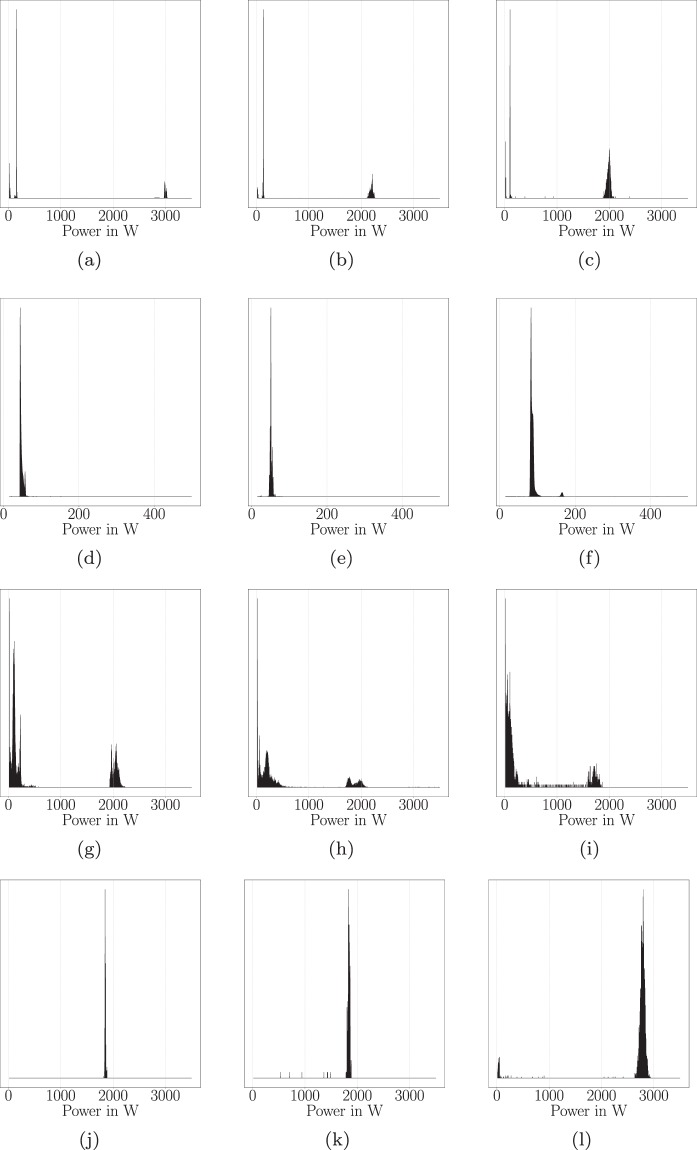
PMFs created from forty days of data: (**a**) dishwasher 1 in SynD, (**b**) dishwasher 2 in ECO, (**c**) dishwasher 2 in UK-DALE, (**d**) fridge 1 in SynD, (**e**) fridge 1 in ECO, (**f**) fridge 2 in REFIT, (**g**) washer 1 in SynD, (**h**) washer 1 in UK-DALE, (**i**) washer 1 in DRED, (**j**) water kettle 1 in SynD, (**k**) water kettle 2 in ECO, (**l**) water kettle 8 in REFIT.

Besides total energy consumption, appliances differ in power states and power consumption patterns i.e. level of power consumption over time. Particularly when evaluating synthetic data, the question arises how similar those synthetic time series are compared to time series that stem from real measurements. In order to answer this question, and to validate our simulation approach, we present a case study that utilises statistical distance measures to quantify the similarity between household appliances from DRED, ECO, REFIT, SynD, and UK-DALE. Our study uses the same household appliances as previous case studies and data from the same forty days. As a first step, we extract the time series for dishwashers, fridges, washing machines, and water kettles from the datasets. Where possible, we extract the time series from the same time of the year (i.e. same months). Next, we clean the time series and resample to a sampling interval of 10 s. Then, we derive the probability mass function (PMF) from the respective time series as described in^31^. We provide examples for some of the derived PMFs in Fig. 7. To enhance readability in those plots, we drop values for power values smaller than 10 W. For the four appliance types considered in Fig. 7, we observe that in comparison to PMFs derived from real data, the PMFs obtained from synthetic data scatter less. However, we identify strong similarity between PMFs of the same appliance category. For instance, the PMFs of the dishwashers all have three representative power states, two below 250 W and one close to 2000 W or above. Similar observations can be made for fridges, washing machines, and water kettles in this study.

To quantify the similarity between synthetic and real appliances, we compute statistical distance measures between probability mass functions. In this study, we use the Hellinger distance and a distance measure based on the Jensen-Shannon divergence. The Hellinger distance^32^ is defined as the Euclidean norm of the difference of the square-roots of two discrete probability distributions P and Q:

3 $$\begin{array}{lll}{D}_{H}(P\parallel Q) & = & \frac{1}{\sqrt{2}}\cdot \sqrt{\sum _{x\in X}{(\sqrt{P(x)}-\sqrt{Q(x)})}^{2}}\\  & = & \frac{1}{\sqrt{2}}\cdot {\parallel \sqrt{P}-\sqrt{Q}\parallel }_{2}\end{array}$$

**Fig. 8:**
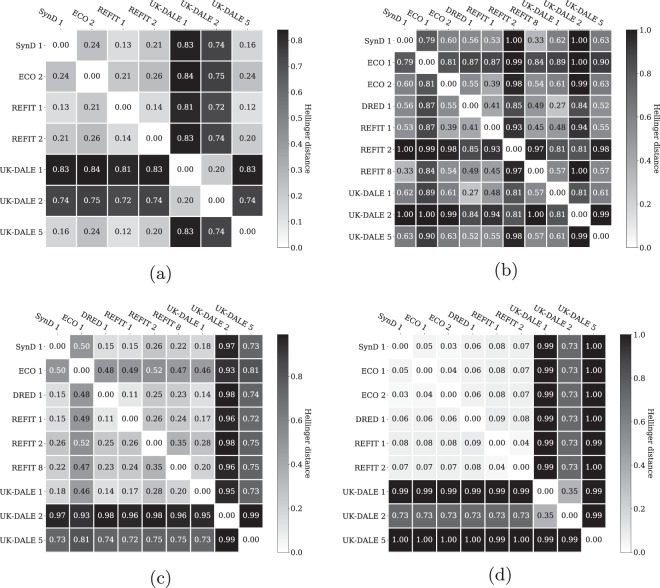
Hellinger distance of probability mass functions for selected appliances: (**a**) dishwashers (**b**) fridges (**c**) washing machines (**d**) water kettles.

A Hellinger distance of 0 indicates total similarity, whereas the maximum value is 1. We derive the Hellinger distance between PMFs of the dishwashers, fridges, washing machines, and water kettles. Figure 8 reports the results of our study. We present four matrices, where one matrix is associated with one appliance type a time. The presented matrices state the similarity in form of the Hellinger distance between two appliances. For every row of a matrix, we compute the Hellinger distance of one appliance, for instance a dishwasher, to all other appliances of the same kind. It should be noted that the diagonal of the matrix is always zero since it reports the distance of a PMF to itself.

We observe low Hellinger distances, *D*_*H*_ < 0.25, between the dishwasher of SynD and dishwashers in ECO 2, REFIT 1, REFIT 2, and UK-DALE 5. In addition, these appliances show pairwise low Hellinger distances, which have approximately the same magnitude as Hellinger distances of SynD. In contrast to that, we measure extraordinarily high distances between the dishwashers of UK-DALE 1, UK-DALE 2 and the remaining dishwashers in our study. Interestingly, UK-DALE 1 and UK-DALE 2 show a Hellinger distance of 0.20. For the fridges in our study, we observe predominantly intermediate as well as high Hellinger distances between the PMFs. Except for rare pairwise exceptions, such as the distance between SynD 1 and REFIT 8, we mostly observe indications of dissimilarity. We observe a large group of washing machines with a low Hellinger distance in our study. The washers in SynD 1, DRED 1, REFIT 1, REFIT 2, REFIT 8, and UK-DALE 1 all show values below 0.35. For ECO 1, we record intermediate similarity to this group of washing machines and large dissimilarity between ECO 1 and UK-DALE 2 as well as UK-DALE 5. In case of water kettles, we identify two major groups: water kettles of UK-DALE and others. Between water kettles of UK-DALE and kettles from other datasets, we measure high Hellinger distances *D*_*H*_ > 0.70. In many cases, we measure maximum dissimilarity. In contrast to that, we observe high similarities between water kettles of SynD, ECO, DRED, and REFIT (*D*_*H*_ < 0.10).

To complement our study, we apply the Jensen-Shannon distance as a second statistical measure to evaluate the similarity of the PMFs. The Jensen-Shannon distance is defined as the square-root of the Jensen-Shannon divergence^33^. This distance measures the similarity between two probability distributions P and Q:

4 $${D}_{JS}(P| | Q)=\sqrt{\frac{1}{2}\cdot ({D}_{KL}(P| | M)+{D}_{KL}(Q| | M))}$$

where M is defined as the point-wise mean of P and Q:

5 $$M=\frac{1}{2}\cdot (P+Q)$$

This distance measure is based on the Kullback-Leibler divergence, is symmetric and always returns a finite value^34^. The Kullback-Leibler divergence^35^, often referred to as relative entropy, is the expectation of the logarithmic difference between P and Q, where the expectation is taken with regard to the probabilities of P:

6 $${D}_{KL}(P| | Q)=\sum _{x\in X}P(x)\cdot log\left(\frac{P(x)}{Q(x)}\right)$$

**Fig. 9:**
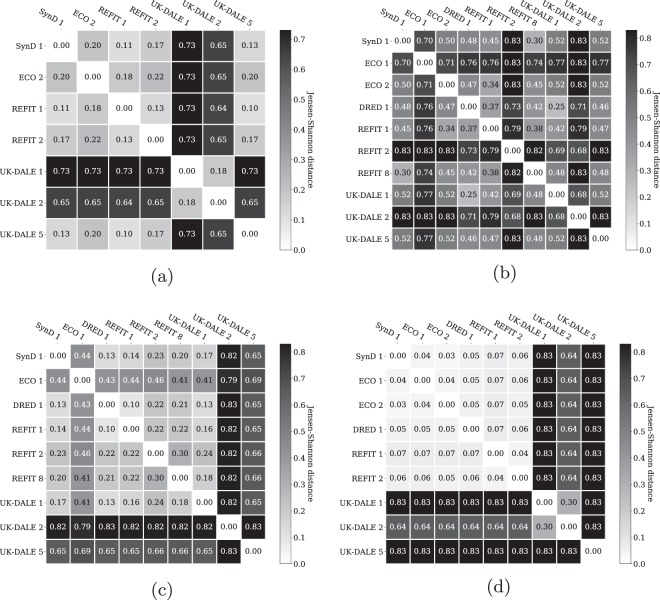
Jensen-Shannon distance of probability mass functions for selected appliances: (**a**) dishwashers (**b**) fridges (**c**) washing machines (**d**) water kettles.

In the same manner as for the Hellinger distance, we derive the Jensen-Shannon distance for PMFs of dishwashers, fridges, washing machines, and water kettles. Figure 9 summarises the JS distance in the form of four matrices, where we form one matrix per appliance type. The obtained matrices closely resemble the outcome of studies related to the Hellinger distance. Based on these results, we draw identical conclusions about pairwise similarities of appliances, we identify the same appliance groups based on pairwise similarity, and we observe that appliances of the dataset UK-DALE show higher degrees of dissimilarity in general.

As concerns statistical similarity in form of the Hellinger or the Jensen-Shannon distance, we identify high degrees of similarity of simulated appliances in SynD and appliances of real-world energy consumption datasets. In addition, we find high levels of pairwise similarity between certain datasets as well as extraordinarily low similarities between other real datasets.

## Discussion

We conclude this section with a summary of our technical validation studies and briefly discuss some limitations of our approach. To demonstrate the technical validity of the synthetic dataset SynD, we present several case studies that evaluate the similarity of SynD and four other energy datasets, which stem from measurement campaigns in real households.

- We demonstrate that the *variation* of the household’s daily energy consumption lies within a realistic range. In some cases, we identified a noticeable smaller variation for real households than for SynD.
- We derived the average load profiles of households for forty days and examined the *spread of appliance usage* during the day. For SynD, we identify resemblance to certain real households but also diagnose limitations of our approach.
- During studies with focus on individual appliances, we find that appliances in SynD show comparable *energy consumption* as real household appliances for an observation period of forty days.
- We derive probability mass functions of selected appliances. Based on those PMFs, we illustrate similarities between real and simulated appliances by help of statistical similarity measures such as Jensen-Shannon distance and Hellinger distance.

The current version of SynD faces certain limitations, which are the result of cost constraints with regard to the measurement campaign or a consequence of our modelling approach:

- Although funds were available to invest in certified measurement hardware, the acquired hardware allowed monitoring single-phase appliances only. Consequently, our measurement campaign excluded big consumers such as electric water heaters, electric three-phase stoves, etc.
- The current version of SynD derives the mains signal by aggregating individual appliance-level power signals. Aggregate power signals of real households contain certain levels of data noise that stems from unmetered appliances, which increases the complexity of the load disaggregation problem^6^. One approach to overcome this limitation could be to superimpose correlated as well as uncorrelated data noise.
- Our approach considers active power only. We hypothesise that incorporating further physical quantities such as apparent power, current, or voltage would increase the value of a synthetic dataset generator for NILM.

## Usage Notes

To help users get started with SynD, we provide a simple code example to demonstrate how to access data. We recommend the use of NILMTK in conjunction with SynD. In principle, working with SynD does not differ from working with other datasets that use the NILMTK data format. To read data from SynD, users have to create a new *DataSet* object and reference the HDF5 file. This object serves to access data and also offers metadata. SynD contains one meter group, *elec*. With the help of this elec object, users can directly access data of the mains or individual appliances. In the code example presented in Box 2, we create a DataSet and an elec object for SynD, print members of the meter group elec, and then plot the aggregate power signal for the household. Further material can be obtained from our repository (https://github.com/klemenjak/synd/).

## Data Availability

We selected Python 3 as main programming language and identify the following dependencies of SynD: Pandas 0.22, Numpy 1.15, and NILMTK 0.3. We aimed at providing compatibility to the latest versions of these software packages and released code examples, an extensive user guide, and supplemental material under the licence Attribution 4.0 International (https://creativecommons.org/licenses/by/4.0/) on our GitHub repository (https://github.com/klemenjak/synd/). Along with the dataset SynD, we release the first public version of our dataset generator tool via figshare^22^. This tool was used to create SynD and can also serve to generate new datasets on-demand. We release this early version of our tool under the licence CC0 (https://creativecommons.org/publicdomain/zero/1.0/).
